# The psychological symptoms and behavioral problems of children with mothers working as medical staff in the crisis of Covid-19 outbreak in Hamadan, Iran

**DOI:** 10.3389/fpsyt.2023.1117785

**Published:** 2023-07-27

**Authors:** Roya Raeisi, Shakiba Gholamzad, Mansoureh Kiani Dehkordi, Mehri Rezaei Kheirabadi, Ali Hhasanpour Ddehkordi, Mohammad Mahdi Sobhani, Mahsa Movahedi

**Affiliations:** ^1^Department of Pediatrics, Hamadan University of Medical Sciences, Hamadan, Iran; ^2^Student Research Committee, Iran University of Medical Sciences, Tehran, Iran; ^3^Department of Psychiatry, Psychosis Research Center, University of Social Welfare and Rehabilitation Sciences, Tehran, Iran; ^4^Department of Psychology, Faculty of Education and Psychology, Isfahan University, Isfahan, Iran; ^5^Hamadan University of Medical Sciences, Hamadan, Iran; ^6^Social Determinants of Health Research Center, School of Allied Medical Sciences, Shahrekord University of Medical Sciences, Shahrekord, Iran; ^7^Student Research Committee, Hamadan University of Medical Sciences, Hamadan, Iran

**Keywords:** COVID-19, treatment staff, children, psychological symptoms, behavioral problems

## Abstract

**Background and objectives:**

The purpose of this study was to investigate the psychological symptoms and behavioral problems of children with mothers working as medical staff in the crisis of Covid-19 disease in Hamadan.

**Methods:**

This descriptive causal-comparative study was conducted on all mothers with children aged 6 to 12  years in Hamadan from September 2 to November 29, 2020. In this study, eligible individuals were selected using random sampling and were assigned to two groups of mothers working as the medical staff and the control group. The research instruments included the Child Behavior Checklist (Achenbach) and the Child Symptom Inventory-4 (CSI-4).

**Results:**

The results showed that the mean scores of psychological and behavioral symptoms of children in terms of group membership (group of mothers working in the medical staff and control group) had a significant difference. There was a significant difference between the mean scores of depression and aggression in children of the staff group and the control group meaning that for depression and aggression scores of children of the staff group are higher than children of the control group (*p* < 0.05). There was no significant difference between the mean anxiety scores and there was almost a significant difference between the attention scores of the staff group and the control group (*p* < 0.05).

**Conclusion:**

Children whose mothers worked as medical staff during Covid-19 show more depression, attention, and aggression problems than children whose mothers do not work as medical staff.

## Introduction

The first known human coronavirus infection occurred in early December 2019. This virus first out broke in mid-December 2019 in Wuhan, China, and soon after, it quickly spread throughout the world, including Iran, causing many deaths (1–4).

In the COVID-19 outbreak crisis, there were reports about the prevalence of psychiatric symptoms in all relevant individuals, including patients, medical staff, patient caregivers, and the general public. As a result, psychological prevention seemed essential. According to statistics, in the early variants of the Coronavirus, children had much fewer clinical symptoms of the respiratory tract than adults, and the mortality rate in children was very low. However, as carriers of the Coronavirus with mild clinical symptoms, they had a large share in the epidemiology of the virus (5). Although the prevalence of this virus in children is reportedly low and with different clinical manifestations; its effects, including the working conditions of parents who are members of health care providers and medical staff, have a direct and significant relationship with the health status of family members including children (6, 7).

Factors such as lack of long leave or non-standard leave and working hours are consistently associated with adverse consequences on children’s health. Most studied health outcomes are behavioral and mental health problems of children, which can affect family relationships including the time spent with children, parental supervision, and the parent’s closeness to children and the home environment. Studies have shown that maternal working hours at night, in the evening, or at irregular times increase the risk of behavioral problems in children. Also, late or irregular return of both parents to home has led to a negative impact on the child’s mental health and has been associated with a decrease in the frequency of parent–child interactions (8–10). Such working hours led to reduced parent–child interaction and reduced quality of parenting and family environment. Poor-quality parenting and lack of parent–child interaction (11–17) are associated with problems in children. Studies have shown that children whose parents return home late due to long working hours show behavioral problems such as hyperactivity and or inattention. In addition, non-standard parental work shifts such as night work and irregular working hours can increase the risk of depression, especially among children (8, 12, 13).

In addition to non-standard working hours, other job-related factors affect children’s health. Fatigue due to sleep deprivation and psychological stress associated with inappropriate working conditions lead to lower quality of time spent with children and subsequently affects their upbringing, which eventually leads to more intense behavioral disorders. Also, instability in the family and a sharp increase in the level of maternal anxiety can lead to less parental support and more children’s externalized symptoms such as aggression. In broken families, children may demonstrate more disturbing behaviors and less interaction with parents which can generate behavioral problems to attract parental attention. On the other hand, some children may distant themselves from their parents and show signs of anxiety (14–17).

Learning about parental anxiety through parental role modeling, parental information transfer, and parenting can reinforce children’s anxious behaviors and can also play an important role in increased anxiety in children (18). Regarding the transfer of workplace stress to the family, research shows that anxious parents inadvertently transfer their insecurity and anxiety to their children, which ultimately leads to a variety of unreasonable fears and worries in them (19).

Due to the crisis of the Covid-19 outbreak, healthcare providers report symptoms of anxiety and distress due to difficult working conditions, long working hours, and extreme fatigue as well as worry and fear of transmitting the disease to their relatives, especially children. Families, television images, and decreased interaction with children are expected to increase the prevalence of psychological symptoms and behavioral problems in children in health care providers compared to children whose parents have unrelated occupations.

This study aimed to investigate the psychological symptoms and behavioral problems of children with mothers working as medical staff during the Covid-19 outbreak crisis in Hamadan.

## Methodology

### Research design

An explanatory research design was adopted to carry out this study.

### Sample and setting

The participants of this case–control study were 118 mothers working as medical staff and their children, and 118 non-working mothers and their children in Hamadan. Mothers responded to all the questions on questionnaires from September 2 to November 29, 2020.

Mothers’ age, children’s age, and children’s gender were matched in the 2 groups. Inclusion criteria were having a 6 to 12-year-old child, being a primary caregiver, and completing the study questionnaires online.

### Data collection and measures

The study tools were the following:

1. The Achenbach Child Behavior Checklist: it is one of the parallel forms of The ***Achenbach*** System of Empirically Based Assessment (***ASEBA***) and evaluates the problems of children and adolescents in 8 categories of anxiety/depression, isolation/depression, physical complaints, social problems, thinking problems, attention problems, ignoring rules, and aggressive behavior. In this study the Persian form of the questionnaire was used and subscales of attention problems and aggressive behavior have been used. Regarding Cronbach’s alpha, the overall validity coefficients of Child Behavior Checklist (CBCL) forms was 0.97, and it was 0.94 by retest validity (20).
2. The Child Symptom Inventory-4 (CSI-4): This questionnaire includes subscales of Attention Deficit Hyperactivity Disorder, Stubbornness Disobedience Disorder, Behavioral Disorder, Anxiety Disorder, Mood Disorder, Psychotic Disorder, Pervasive Developmental Disorders, and Excretory Disorders. In this study the Persian form of the questionnaire was used. The reliability of the questionnaire was determined through a retest on 4 diagnostic groups from 0.70 to 0.89 and its validity was reported at 0.80 (21).

### Statistical analysis

Results were summarized as mean ± standard deviation (SD) for quantitative variables and frequency (percentage) for categorical variables. Continuous variables were compared using the t-test or the Mann–Whitney test if the data did not appear to have a normal distribution or if the assumption of equal variances in the study groups was violated. Categorical variables, on the other hand, were compared using chi-square tests. *p* values of ≤0.05 were considered statistically significant. SPSS statistical software version 23.0 for Windows (IBM, Armonk, New York) was used for statistical analysis.

## Results

After removing incomplete questionnaires, 236 subjects (118 mothers working as medical staff and their children, and 118 nonworking control subjects and their children) were included in the study. The two groups were matched for the average age of the mothers, the age of the children, and the gender of the children.

According to the results in Table 1, the average age of mothers working as medical staff is 36.08 and the average age of the control group is 36.89. In addition, the mean age of children in the control group is 6.85, and the mean age of children of medical staff mothers is 7.85 (see Table 1). According to Table 2, the participants in the medical staff mothers group include 45.8% girls and 54.2% boys and the participants in the control group include 55.9% girls and 44.1% boys. According to Table 3, the mean scores of children’s psychological and behavioral symptoms differ between the group of medical staff mothers and the control group. Children in the medical staff mothers group had higher mean scores for depression, attention problems, and aggression as compared to children in the control group; but there was no significant difference between the mean scores of general anxiety among children of medical staff mothers group and the control group. The descriptive indicators of the research variables were illustrated in Table 3. The results show that the mean scores of children’s psychological and behavioral symptoms differ in terms of group membership (medical staff mothers group and a control group). The results of the independent t-test show that there is a significant difference between depression and aggression in the two groups of children of working mothers and the control group (*p* < 0/05). It means that depression and aggression scores of the children of medical staff mothers are higher than children in the control group (*p* < 0.05).

**Table 1:** Mean age of mothers and children according to group membership.

Group		Mean ± SD	*p*.Value
Age of mothers	Mothers Working as medical staff	36.08 ± 6.46	0.408
	Control	36.89 ± 6.58	
Age of children	Mothers Working as medical staff	7.85 ± 4	0.821
	Control	6.85 ± 3.01	

**Table 2:** Gender distribution by group membership.

Group	Frequency (%)		*p*.Value
Mothers Working as medical staff	Girl	54(45.8)	0.067
	Boy	64(54.2)	
Control	Girl	66(55.9)	
	Boy	52(44.1)	

**Table 3:** Descriptive indicators of psychological and behavioral symptoms of children based on the group membership.

Variable	Group	Mean ± SD	Mean difference	*p*.Value^*^
anxiety	Medical staff mothers	13.81 ± 9.31	0.32	0.78
	Control	13.49 ± 8.07		
Depression	Medical staff mothers	2.52 ± 1.89	−0.78	0.01
	Control	3.30 ± 2.53		
Attention	Medical staff mothers	3.36 ± 2.61	−0.80	0.05
	Control	4.16 ± 3.46		
Aggression	Medical staff mothers	7.29 ± 6.02	−2.83	<0.001
	Control	10.12 ± 7.90		

*Results of independent *t*-test to compare the behavioral symptoms and problems of children in terms of mother’s group membership based on the alpha extract from Bonferroni correction $\left(\alpha =0.05/4=0.0125\right).$

## Discussion

The working conditions and the effects on the mental health of the individuals and those around them have been the focus of health research. This study examined and compared behavioral problems and psychological symptoms in children of medical staff mothers during the crisis of COVID-19 outbreak as compared to the control group. Much research has been carried out on the effect of parents’ working conditions on their children’s mental health, such as Han WJ et al., (22) study in China, that found “children whose fathers worked night shifts had internalizing behaviors” (23). However, there has been no research on critical situations such as the outbreak of pandemics, including COVID-19. Given the critical nature and prolongation of the epidemic, the involvement of the medical staff, as parents, and longer working conditions and exacerbated psychological stress, affecting their mental health, research was needed to design intervention projects to improve the mental health of working mothers and their children by assessing the current situation. The results of the present study showed that children with parents working as the medical staff showed problems and symptoms of aggression and depression, and attention problems significantly more than children in the control group whose parents were not working as the medical staff. However, in terms of anxiety symptoms, no significance, and in terms of attention almost significance was observed in these two groups. These results are in line with the research by Kizuki et al., (8) who surveyed 2,987 children and their families in Japan. In their study, they found that children with both parents returning home late or having irregular working hours were more likely to demonstrate behavioral and attention problems. In addition, a study conducted by Vieira et al., (21) on parent-family work experiences and children’s behavioral problems in Portugal showed that parent-work–family conflicts have a positive relationship with children’s externalized problems [aggression, attention, etc., (8, 24)].

According to research, employment, and its conditions have different psychological effects on children’s health. Factors such as lack of leave and long and non-standard working hours are consistently associated with adverse outcomes in children’s health (6, 10). We cannot determine the exact mechanism of these relationships through the results of our research, but some possible explanations can be as follows. In the current critical situation, long and irregular working hours lead to less interaction between parents and children and lower quality of parenting and the family environment. These non-standard working conditions and working hours have an independent impact on the child’s mental health. Also, children whose parents returned home late due to long working hours had behavioral problems and inactivity/inattention. In addition, children whose mothers did shift work were more likely to exhibit delinquent behaviors and behavioral problems than others. These behaviors were mainly surfaced at school. In addition, non-standard parental work shifts such as night shifts and irregular working hours can increase the risk of inattention followed by aggression among children (8, 9, 11, 13, 22).

Mental health symptoms may have been common during the COVID-19 outbreak among the general population, especially among infected individuals, people with suspected infection, and people who might have contact with patients with COVID-19. Some measures, such as quarantine and delays in returning to work, had been also associated with mental health of the public (25).

These results are consistent with the results of our study. In addition, anxiety is another issue that children with parents having stressful jobs might develop. Research on the transfer of workplace stress to the family in the city of Kerman, Iran has shown that anxious parents inadvertently transmit their insecurity and anxiety to children, which ultimately leads to a variety of unreasonable fears and worries (19). However, the results of the present study indicate that there is no significant difference in the anxiety of children in the two groups. In some studies, such as the one by Vieira et al., (21) they found that non-standard working conditions and long and variable shifts were directly related to externalized problems in children (such as aggression, disobedience, and attention problems); however, they had no association with internalized problems such as emotional symptoms of anxiety. This could be because work–family conflicts also lead to the exaggeration of externalized problems in children through negative effects on the quality of the parent–child relationship, but these problems do not contribute to the development of internalization. Evidence shows that reduced parental control over children, which includes frequent interactions and conversations with children about their activities and friends, leads to loneliness in children and externalized behavioral problems such as aggression, attention problems, and hyperactivity. These externalized behavioral problems are some ways to attract the attention of parents (24, 26, 27). Therefore, the parents of the medical staff are not able to interact positively and adequately with their children, due to having long and irregular working hours, night shifts, and exhaustion during the outbreak of Corona crisis, as well as critical conditions and worries for those around. This has led to an increase in children’s feelings of loneliness, followed by behavioral problems such as aggression and attention problems, and perhaps this loneliness and lack of communication between parents and other friends and relatives have led to parents cannot convey much of the anxiety caused by the work environment and critical situations to their children.

## Conclusion

This study examined and compared behavioral problems and psychological symptoms in children of medical staff during COVID-19 outbreak crisis and a control group including children of mothers with unrelated jobs. The results showed that the children in the group of medical staff mothers had higher mean scores for depression and aggression than the children in the control group. However, there was no significant difference between the mean scores for general anxiety, and there was almost a significant difference between the mean scores for attention among the children in the group of medical staff mothers and the control group.

Although it seems that the special working conditions of the medical staff lead to their work and family conflicts and these conflicts leave negative effects on the children, in some ways, it can be pointed out that these children experience a different lifestyle from a younger age. So, they show more psychological compatibility in some aspects of life. Also, job conditions increase the tolerance threshold of mothers and make them more adaptable to specific work and family conditions, which reduces the transmission of anxiety from mothers to children. It is suggested that this study should be done in other organizations with an emphasis on the issue of children of mothers with special job conditions. In addition to the negative effects, its positive effects should also be addressed and analyzed so that appropriate solutions be presented. Also, educational and work–family conflict management workshops should be held in hospitals and other medical centers. In addition, it is suggested to conduct this research in older age groups.

## Data availability statement

The original contributions presented in the study are included in the article/supplementary material, further inquiries can be directed to the corresponding authors.

## Ethics statement

The studies involving human participants were reviewed and approved by Hamadan University of Medical Sciences, Hamadan, Iran. Written informed consent to participate in this study was provided by the participants' legal guardian/next of kin (IR.UMSHA.REC.1402.155).

## Author contributions

RR, SG, MRK, AHD, and MKD conception and design of the work, also the acquisition, analysis, and interpretation of data for the work. AND drafting the work and agreement to be accountable for all aspects of the work in ensuring that questions related to the accuracy or integrity of any part of the work are appropriately investigated and resolved. RR, SG, MRK, AHD, and MKD revising final approval of the version to be published. All authors contributed to the article and approved the submitted version.

## Conflict of interest

The authors declare that the research was conducted in the absence of any commercial or financial relationships that could be construed as a potential conflict of interest.

## Publisher’s note

All claims expressed in this article are solely those of the authors and do not necessarily represent those of their affiliated organizations, or those of the publisher, the editors and the reviewers. Any product that may be evaluated in this article, or claim that may be made by its manufacturer, is not guaranteed or endorsed by the publisher.
